# Between-session reliability of performance and asymmetry variables obtained during unilateral and bilateral countermovement jumps in basketball players

**DOI:** 10.1371/journal.pone.0255458

**Published:** 2021-07-30

**Authors:** Alejandro Pérez-Castilla, Amador García-Ramos, Danica Janicijevic, Gabriel Delgado-García, Juan Carlos De la Cruz, F. Javier Rojas, Mar Cepero

**Affiliations:** 1 Faculty of Sport Sciences, Department of Physical Education and Sport, University of Granada, Granada, Spain; 2 Faculty of Education, Department of Sports Sciences and Physical Conditioning, Universidad Católica de la Santísima Concepción, Concepción, Chile; 3 Faculty of Sport and Physical Education, University of Belgrade, The Research Centre, Belgrade, Serbia; 4 Faculty of Educational Sciences, Department of Didactics of Musical, Plastic and Corporal Expression, University of Granada, Granada, Spain; Universidade Federal de Mato Grosso do Sul, BRAZIL

## Abstract

This study aimed to evaluate the between-session reliability of single-leg performance and asymmetry variables during unilateral and bilateral countermovement jumps (CMJ). Twenty-three basketball players completed two identical sessions which consisted of four unilateral CMJs (two with each leg) and two bilateral CMJs. Mean and peak values of force, velocity and power, impulse, and jump height were obtained separately for each leg using a dual force platform. All performance variables presented an acceptable reliability (CV_range_ = 4.05–9.98%) with the exceptions of jump height for the unilateral CMJs and mean power, peak velocity, peak power, and impulse for the left leg during the bilateral CMJ (CV≥11.0%). Nine out of 14 variables were obtained with higher reliability during the unilateral CMJ (CV_ratio_≥1.16), and 4 out of 14 during the bilateral CMJ (CV_ratio_≥1.32). Asymmetry variables always showed an unacceptable reliability (ICC_range_ = 0.15–0.64) and *poor*/*slight* levels of agreement in direction (Kappa_range_ = -0.10 to 0.15) for the unilateral CMJ, while an acceptable reliability (ICC_range_ = 0.74–0.77) and *substantial* levels of agreement in direction (Kappa_range_ = 0.65 to 0.74) were generally obtained for the bilateral CMJ. These results suggest that single-leg performance can be obtained with higher reliability during the unilateral CMJ, while the bilateral CMJ provides more consistent measures of inter-limb asymmetries.

## Introduction

The countermovement jump (CMJ) is a ballistic exercise commonly performed on a force platform to comprehensively assess lower-body neuromuscular function [1,2]. The CMJ has been also used to measure single-leg performance and detect inter-limb asymmetries [3–5]. The assessment of inter-limb asymmetries is justified by the influence that CMJ-based asymmetry may have on athletic performance [6,7] and injury risk [4]. Both unilateral and bilateral CMJs have been used to detect inter-limb asymmetries [3–6,8]. During unilateral CMJs athletes jump from a monopodial stance with the tested leg placed on a single force platform, while during the bilateral CMJ a bipodal stance is used with each leg positioned on an individual force platform [5]. Although it is reasonable to believe that the inter-limb asymmetries measured during bilateral and unilateral CMJs should be closely related, research has shown that impulse asymmetries observed during bilateral and unilateral CMJs are unrelated [3]. In fact, it has been suggested that the unilateral CMJ represents a more robust indicator of the capacity of each limb, while the bilateral CMJ may provide more comprehensive information about the compensatory strategies between limbs [9]. Therefore, it is important to elucidate whether bilateral or unilateral CMJs provides more valuable data to accurately detect inter-limb asymmetries [10].

A high reliability is a basic requirement for any fitness test [11]. Emerging research has explored the reliability of single-leg mechanical performance variables collected with force platforms during unilateral and bilateral CMJs. Regardless of the tested leg, some studies have reported a high between-session reliability (coefficient of variation [CV] ≤ 9.8%; intraclass correlation coefficient [ICC] ≥ 0.75) for peak force, peak power, concentric impulse, and jump height during the unilateral CMJ [12–18]. Similar reliability outcomes were reported for the lower-limb differences in mean force, peak force, and concentric impulse during the bilateral CMJ performed with and without arm swing (CV ≤ 9.2%; ICC ≥ 0.68) [5,19]. The available body of literature suggests that both CMJ variants could be reliable tests to measure single-leg mechanical performance. Instead, Benjanuvatra et al. [3] have recommended the unilateral CMJ to examine impulse asymmetries because it places a greater emphasis on force production from one limb, while inter-limb asymmetries detected during the bilateral CMJ could be more affected by the weighing phase (i.e., slight shifts of the center-of-mass toward one side). It is worth mentioning that the purpose of the weighing phase is the accurate determination of body weight, which is essential in forward dynamics procedures [20]. Contrary to Benjanuvatra’s et al. [3] suggestion, a recent study [19] observed that the single-leg performance for the mean force (CV_ratio_ = 1.29–1.41), concentric impulse (CV_ratio_ = 1.56–1.63), and eccentric impulse (CV_ratio_ = 1.45–1.75) was more reliable for the bilateral CMJ than unilateral CMJ in elite academy soccer players. Therefore, this lack of agreement clearly emphasizes the need to conduct additional research to elucidate which variant of CMJ exercise is more reliable to measure single-leg performance considering other mechanical variables (e.g., velocity, power, or jump height) and populations (e.g., basketball players).

On the other hand, only two studies [3,4] have examined the between-session reliability of CMJ-based asymmetry variables. Impellizzeri et al. [4] showed that *“Bilateral Strength Asymmetry”* was highly reliable for peak force (ICC = 0.91) measured with a single force platform (i.e., athletes jump with one leg placed on a force platform and the other leg on a wooden platform). Benjanuvatra et al. [3] also found that the *“Index of Asymmetry”* calculated for jump height and concentric impulse presented a high reliability (ICC ≥ 0.95) during bilateral and unilateral CMJs. The scope of other studies, however, focused on answering the question of whether asymmetry consistently favored the same leg between testing sessions (i.e., *“direction of asymmetry”*) during unilateral CMJ [8,13,19]. For instance, Bishop et al. [13] found *substantial* levels of agreement (Kappa range = 0.64–0.66) for the peak force and jump height collected with a single force platform in recreational soccer and rugby athletes. By contrast, Bishop et al. [8] also reported a *slight* to *fair* levels of agreement (Kappa range = 0.18–0.29) for the jump height measured with the smartphone application *“My jump 2”* in national-level youth basketball athletes. Therefore, further research is warranted to examine the between-session reliability of inter-limb asymmetry variables during unilateral and bilateral CMJs in order to ensure that the magnitude and direction are consistent between sessions [21].

To address the gaps in the literature, we assessed different mechanical variables separately for the left and right legs using a dual force platform during the unilateral and bilateral CMJ variants, and thereafter calculated inter-limb asymmetries. Specifically, the aim of this study was to elucidate whether single-leg performance and inter-limb asymmetries can be obtained with a higher reliability during unilateral or bilateral CMJ variants. It was hypothesized that the reliability of single-leg performance variables would be higher for the unilateral CMJ compared to the bilateral CMJ due to an expected higher variability in the weighing phase for the bilateral CMJ [3]. Regarding the comparison of the reliability of inter-limb asymmetries between the unilateral and bilateral CMJ variants, no specific hypothesis was formulated due to the contrasting findings regarding the unilateral CMJ [8,13] and the lack of straightforward evidence for the bilateral CMJ.

## Material and methods

### Subjects

Twenty-three amateur basketball players volunteered to participate in this study. Specifically, the study sample was composed of a senior male (n = 11; age = 19.2 ± 1.5 years [range: 17–22 years]; body mass = 79.3 ± 11.0 kg; body height = 1.87 ± 0.08 m) and female (n = 12; age = 21.1 ± 4.2 years [range: 15–29 years]; body mass = 70.6 ± 7.2 kg; body height = 1.75 ± 0.06 m) team that played in a regional-level Spanish basketball club (data presented as mean ± standard deviation [SD]). All subjects had at least five years of competitive experience and were accustomed to performing the unilateral and bilateral CMJ exercises as part of their habitual strength and conditioning training routines. No physical limitations, health problems or musculoskeletal injuries that could compromise testing were reported. Prior to testing, subjects were informed about the research purpose and procedures, and they or their legal guardians (for subjects aged < 18 years) gave written consent to participate in the study. The study protocol adhered to the tenets of the Declaration of Helsinki and was approved by the University of Granada Institutional Review Board (IRB approval: 1706/CEIH/2020).

### Experimental design

A repeated-measures design was used to compare the between-session reliability of single-leg mechanical performance and inter-limb asymmetry variables between unilateral and bilateral CMJs. Subjects completed two identical sessions separated by seven days. Each testing session consisted of four unilateral CMJ (two trials with each leg) and two bilateral CMJs. The order of the CMJ variants was randomized in session 1, but in session 2 subjects were assigned with the same order as in session 1. Only the trial with higher jump height of each session for each CMJ variant was used for statistical analyses. All testing sessions were performed at the same facility, under the direct supervision of the same experimenter, and were held between 19:00–21:00 hours. Subjects continued with their regular training program over the course of the study, but they were asked to refrain from any strenuous physical activity for at least 24 hours prior to testing days.

### Testing procedures

Body height and body mass were measured at the beginning of the first session using a wall-mounted stadiometer (Seca 202 Stadiometer; Seca Ltd., Hamburg, Germany) and an eight-electrode system (Tanita BC-418 MA; Tanita Corp., Tokyo, Japan), respectively. The warm-up consisted of 5 minutes of jogging, lower-limb dynamic stretching exercises, and three sub-maximal practice trials of each CMJ variant. The jogging pace and lower-limb dynamic stretching exercises were self-selected by the subjects as they commonly do in their usual training. After warming-up, subjects rested 3 minutes and then they performed two trials for each CMJ variant. The order of execution of the CMJ variants was randomized in the first session, and the same order was followed in the second session. The rest between trials of the same and different CMJ variants was set to 1 and 2 minutes, respectively. The specific characteristic of the unilateral and bilateral CMJ variants were the following.

#### Unilateral CMJ

Subjects began the exercise execution by standing in an unilateral stance with the tested leg fully extended and placed on the center of a force platform, the alternate leg flexed to 90° at the hip and knee joints, and the hands placed on the hips [22]. Subsequently, subjects were instructed to jump as high as possible and to land on the same tested leg after performing a countermovement to a self-selected depth. Subjects had to keep their hands on their hips throughout the movement and the tested leg remained fully extended during the flight phase. The swing of the opposite leg prior to the jump was prohibited [23]. An experienced examiner asked the subjects to repeat the trial after 1 minute of rest when the jump did not comply with these instructions.

#### Bilateral CMJ

Subjects began the exercise execution by standing in a comfortable bilateral stance with each leg fully extended and feet hip-width apparated and positioned over the center of two parallel force platforms, and with the hands placed on the hips [24]. The execution technique was identical to the unilateral CMJ, with the difference that subjects were instructed to jump and land on both legs simultaneously.

### Measurement equipment and data analysis

All CMJ tests were performed on two parallel force platforms (Type 9260AA6; Kistler, Winterthur, Switzerland) embedded in a wooden drawer. The vertical ground reaction force (vGRF) data from each force platform were synchronously acquired via BioWare^®^ software (Kistler, Winterthur, Switzerland) at a sampling rate of 1,000 Hz. The force platforms were zeroed before each trial. The vGRF data were exported as text files and analyzed using a customized 2019 Microsoft Excel^®^ spreadsheet (version 16.32, Microsoft Corporations, Redmond, Washington, USA) [25].

During the weighing phase, subjects stood still on one (unilateral CMJs) or two (bilateral CMJ) legs for approximately 3 seconds. Body weight and the *SD* of the weighing phase were determined during the last second preceding the onset of the countermovement [26]. The countermovement phase started 30 ms before the instant in which the vGRF was lower than the body weight minus 5 *SD* of the weighing phase [27] and finished when the velocity of the center-of-mass was positive [25]. The propulsion phase was identified from this latter point until the take-off instant. The take-off and landing were determined in three steps [22,25]: (I) identification of the first force value lower than 10 N and the next force value greater than 10 N, (II) selection of 100 ms for unilateral CMJ or 300 ms for bilateral CMJ between the points identified in stage I, and (III) calculation of the mean vGRF and *SD* of the time frame representing the flight phase identified in stage II. Thereafter, the take-off and landing instants were determined as the first force value lower or greater than the mean vGRF plus 5 *SD* of the flight phase, respectively.

The impulse-momentum approach was used to calculate the dependent variables of the present study [28]. Vertical acceleration was calculated as the net vGRF divided by body mass, while vertical velocity of the center-of-mass was determined by integrating acceleration with respect to time. Vertical power was calculated as the product of force and velocity at each time point. The mean and peak values of force, velocity, and power, as well as the net vertical impulse were calculated during the propulsive phase of the jump (in the further text this variable will be referred to as concentric impulse). Finally, the jump height was estimated from the flight time using the following equation [28,29], where *g* represents gravity acceleration (-9.81 m·s^-2^): jump height = g*(flight time)^2^/8.

### Statistical analyses

Descriptive data are presented as means, SD, and range. The normal distribution of the data was confirmed using the Shapiro-Wilk test (*P* > 0.05; except for the magnitude of the mean velocity for the right leg and jump height for the left leg during unilateral CMJs, and inter-limb asymmetries in peak power, peak velocity and concentric impulse during the unilateral CMJ). Paired samples *t*-tests for normally distributed variables or the Wilcoxon signed-rank test for non-normally distributed variables, in addition to the standardized mean difference (Cohen’s *d* effect size [ES]), were used to compare the magnitude of the performance and inter-limb asymmetry variables between both testing sessions. The criteria to interpret the magnitude of the ES was the following: *trivial* (<0.20), *small* (0.20–0.59), *moderate* (0.60–1.19), *large* (1.20–2.00), or very large (>2.00) [30]. Absolute (CV% = standard error of measurement/subjects’ mean score × 100) and relative reliability (ICC, model 3.1) were calculated with their corresponding 95% confidence intervals. Acceptable reliability was determined as an ICC > 0.70 and CV < 10% [31]. The ratio between two CVs (higher CVvalue/lower CV value) was used to compare the between-session reliability of performance variables between unilateral and bilateral CMJs. The smallest important ratio between two CVs was considered to be higher than 1.15 [32].

Standard percentage differences (100/[maximum value from right and left leg]*[minimum value from right and left leg]*[–1] + 100) were calculated to assess inter-limb asymmetries during the unilateral CMJ [33]. The bilateral asymmetry index-1 ([preference leg–non-preference leg]/[preference leg + non-preference leg]*100) was used to assess inter-limb asymmetries during the bilateral CMJ [33]. To determine the direction of asymmetry during the unilateral CMJ, an “IF function” (IF*[left leg < right leg, 1, -1]) was added to the end of the asymmetry equation [34]. Leg preference during the bilateral CMJ was determined via questionnaire (2 males and 1 female were left-footed) [35,36]. Finally, kappa coefficients were calculated to determine the levels of agreement for the direction of the asymmetries between both testing sessions [13]. For that, data were first coded on a subject-by-subject basis; where the direction of asymmetry was assigned as “1” when favored the right leg (unilateral CMJ)/preference (bilateral CMJ) or “0” when favored the left leg (unilateral CMJ)/non-preference (bilateral CMJ). The criteria to interpret the kappa values were as follows: *poor (≤ 0*.*00)*, *slight* (0.01–0.20), *fair* (0.21–0.40), *moderate* (0.41–0.60), *substantial* (0.61–0.80), or *almost perfect* (0.81–0.99) [37]. All reliability assessments were performed by means of a custom Excel spreadsheet [38], while other statistical analyses were performed using the software package SPSS (IBM SPSS version 22.0, Chicago, IL). Alpha was set at 0.05.

## Results

**Fig 1:**
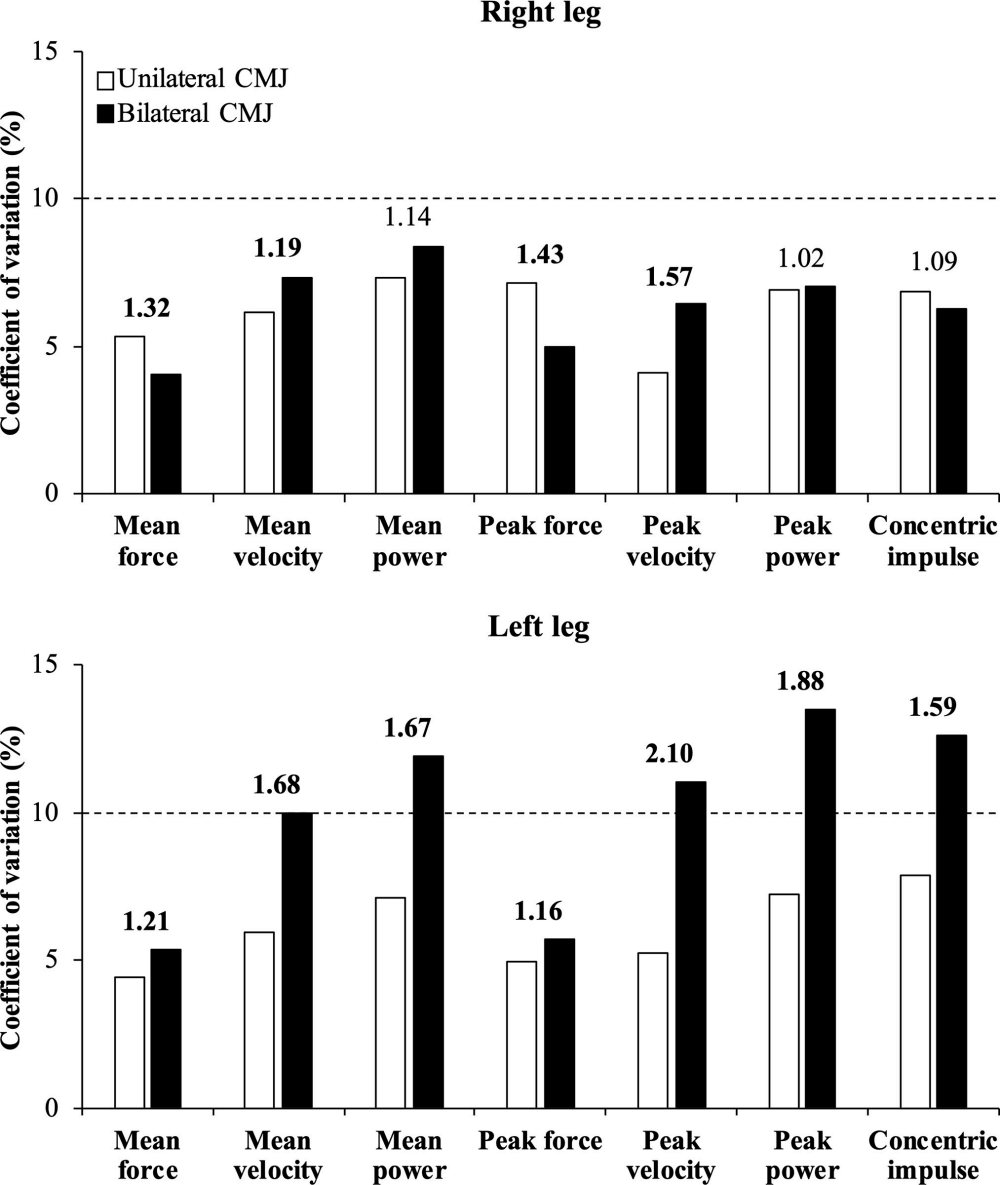
Comparison of the absolute reliability of the different performance variables obtained with the right (upper panel) and left (lower panel) legs between the unilateral (white bars) and bilateral (black bars) countermovement jump (CMJ) exercises. Numbers depict the ratio between two coefficients of variation (CV_ratio_ = higher value/lower value), while meaningful differences in reliability are indicated in bold (CV_ratio_ > 1.15).

Descriptive data of single-leg performance and inter-limb asymmetry variables are presented in Table 1. No significant differences between both testing sessions were observed for most of the performance variables (*P* > 0.05; 21 out of 30 comparisons), while the magnitude of the differences was either *trivial* (ES ≤ 0.19; 24 out of 30 comparisons) or *small* (0.20 ≤ ES ≤ 0.29; 6 out of 30 comparisons). All performance variables presented an acceptable reliability (CV range = 4.05–9.98%; ICC range = 0.82–0.97) with the exceptions of jump height for both unilateral CMJs and mean power, peak velocity, peak power, and concentric impulse for the left leg during the bilateral CMJ (CV ≥ 11.0%) (Table 2). Regarding the reliability comparison between the CMJ variants, the unilateral CMJ reported a greater reliability (CV_ratio_ range = 1.16–2.10) in 2 out of 7 comparisons for the right leg (mean and peak velocity) and all comparisons for the left leg (mean and peak values of force, velocity, power, and concentric impulse) compared to the bilateral CMJ, while the bilateral CMJ was more reliable (CV_ratio_ range = 1.32–1.43) in 2 out of 7 comparisons for the right leg (mean and peak force) (Fig 1).

**Table 1:** Descriptive data of performance and inter-limb asymmetry variables obtained during the unilateral and bilateral countermovement jump (CMJ) exercises.

Variable	Session	Unilateral CMJ			Bilateral CMJ		
		Right leg	Left leg	Asymmetry (%)	Right leg	Left leg	Asymmetry (%)
Mean force (N)	1	1061 ± 159	1051 ± 162	0.9 ± 7.7	661 ± 104	653 ± 91	-0.4 ± 3.9
	2	1082 ± 163	1099 ± 173	-1.4 ± 4.7	664 ± 102	660 ± 91	0.4 ± 3.7
Mean velocity (m·s^-1^)	1	1.04 ± 0.18	1.04 ± 0.16	-0.2 ± 8.3	1.40 ± 0.41	1.38 ± 0.41	5.7 ± 22.6
	2	1.08 ± 0.19	1.06 ± 0.18	2.3 ± 9.3	1.49 ± 0.42	1.34 ± 0.41	5.5 ± 24.9
Mean power (W)	1	1037 ± 271	1032 ± 249	0.0 ± 12.4	880 ± 340	835 ± 285	5.7 ± 24.6
	2	1093 ± 286	1083 ± 299	0.9 ± 10.3	937 ± 364	817 ± 295	5.8 ± 27.5
Peak force (N)	1	1306 ± 196	1275 ± 208	2.4 ± 6.9	795 ± 124	785 ± 107	-0.5 ± 3.6
	2	1326 ± 226	1328 ± 229	-0.1 ± 5.3	810 ± 124	805 ± 115	0.1 ± 2.9
Peak velocity (m·s^-1^)	1	1.86 ± 0.27	1.87 ± 0.24	-0.7 ± 8.6	2.53 ± 0.71	2.48 ± 0.77	5.9 ± 23.9
	2	1.88 ± 0.29	1.84 ± 0.27	1.8 ± 6.7	2.68 ± 0.76	2.39 ± 0.69	5.4 ± 24.6
Peak power (W)	1	2048 ± 490	2036 ± 497	0.2 ± 12.8	1670 ± 623	1605 ± 547	5.7 ± 25.2
	2	2079 ± 540	2024 ± 561	2.6 ± 8.0	1782 ± 686	1550 ± 503	5.2 ± 26.2
Concentric impulse (N·s)	1	121.4 ± 29.4	123.4 ± 25.8	-2.7 ± 13.5	89.2 ± 28.0	86.0 ± 26.3	4.3 ± 20.8
	2	120.7 ± 27.0	121.8 ± 28.8	-0.7 ± 9.5	92.9 ± 29.1	82.4 ± 24.7	5.4 ± 21.1
Jump height (m)	1	0.15 ± 0.05	0.16 ± 0.04	-4.9 ± 15.5			
	2	0.16 ± 0.05	0.16 ± 0.05	-3.3 ± 13.3			

Data are presented as means ± *SD*.

**Table 2:** Reliability of performance and inter-limb asymmetry variables obtained during the unilateral and bilateral countermovement jump (CMJ) exercises.

Exercise	Variable	Right leg				Left leg				Asymmetry (%)		
		*P*	ES	CV (95% CI)	ICC (95% CI)	*P*	ES	CV (95% CI)	ICC (95% CI)	*P*	ES	ICC (95% CI)
Unilateral CMJ	Mean force	0.221	0.13	5.34 (4.13, 7.55)	0.88 (0.75, 0.95)	0.003	0.29	4.45 (3.44, 6.29)	0.93 (0.83, 0.97)	0.151	-0.36	**0.34 (-0.07, 0.66)**
	Mean velocity	0.390	0.26	6.17 (4.77, 8.73)	0.88 (0.74, 0.95)	0.306	0.11	5.93 (4.59, 8.39)	0.88 (0.74, 0.95)	0.288	0.29	**0.22 (-0.21, 0.57)**
	Mean power	0.025	0.20	7.34 (5.68, 10.4)	0.93 (0.84, 0.97)	0.032	0.19	7.14 (5.52, 10.1)	0.93 (0.85, 0.97)	0.691	0.08	**0.51 (0.13, 0.76)**
	Peak force	0.480	0.09	7.14 (5.52, 10.1)	0.82 (0.62, 0.92)	0.010	0.24	4.92 (3.80, 6.96)	0.92 (0.83, 0.97)	0.153	-0.40	**0.15 (-0.27, 0.52)**
	Peak velocity	0.415	0.07	4.08 (3.16, 5.78)	0.93 (0.85, 0.97)	0.277	-0.12	5.24 (4.05, 7.42)	0.87 (0.71, 0.94)	0.279	0.32	**0.64 (0.32, 0.83)**
	Peak power	0.469	0.06	6.93 (5.36, 9.81)	0.93 (0.84, 0.97)	0.794	-0.02	7.20 (5.57, 10.2)	0.93 (0.84, 0.97)	0.464	0.22	**0.56 (0.20, 0.79)**
	Concentric impulse	0.786	-0.02	6.87 (5.31, 9.72)	0.92 (0.82, 0.97)	0.599	-0.06	7.90 (6.11, 11.2)	0.89 (0.75, 0.95)	0.571	0.17	**0.49 (0.11, 0.75)**
	Jump height	0.714	0.04	**12.8 (9.91, 18.1)**	0.85 (0.68, 0.93)	0.975	0.02	**11.5 (8.86, 16.2)**	0.86 (0.69, 0.94)	0.638	0.11	**0.42 (0.02, 0.70)**
Bilateral CMJ	Mean force	0.751	0.02	4.05 (3.13, 5.73)	0.94 (0.86, 0.97)	0.532	0.07	5.38 (4.16, 7.61)	0.86 (0.70, 0.94)	0.206	0.19	0.77 (0.53, 0.90)
	Mean velocity	0.009	0.22	7.34 (5.68, 10.4)	0.94 (0.86, 0.97)	0.356	-0.09	9.98 (7.72, 14.1)	0.90 (0.78, 0.96)	0.963	-0.01	0.74 (0.47, 0.88)
	Mean power	0.018	0.16	8.38 (6.48, 11.9)	0.96 (0.90, 0.98)	0.550	-0.06	**11.9 (9.23, 16.9)**	0.89 (0.77, 0.95)	0.976	0.00	0.76 (0.51, 0.89)
	Peak force	0.226	0.12	5.01 (3.87, 7.09)	0.90 (0.79, 0.96)	0.158	0.18	5.72 (4.42, 8.09)	0.85 (0.67, 0.93)	0.352	0.17	**0.63 (0.31, 0.83)**
	Peak velocity	0.004	0.21	6.42 (4.97, 9.09)	0.95 (0.89, 0.98)	0.253	-0.13	**11.0 (8.51, 15.6)**	0.88 (0.73, 0.95)	0.895	-0.02	0.75 (0.50, 0.89)
	Peak power	0.005	0.17	7.05 (5.46, 9.98)	0.97 (0.93, 0.99)	0.393	-0.10	**13.5 (10.4, 19.1)**	0.85 (0.68, 0.93)	0.897	-0.02	0.75 (0.50, 0.89)
	Concentric impulse	0.039	0.13	6.28 (4.86, 8.89)	0.96 (0.92, 0.98)	0.263	-0.14	**12.6 (9.70, 17.8)**	0.84 (0.66, 0.93)	0.734	0.05	0.74 (0.48, 0.88)

*P*, *P*-value obtained through a paired samples *t*-test or Wilcoxon signed-rank test depending whether the variables were or not normally distributed between the sessions 1 and 2; ES = Cohen’s *d* effect size ([Session 2 –Session 1/*SD* both]); CV = coefficient of variation; ICC = intraclass correlation coefficient; 95% CI = 95% confidence interval. Bold numbers indicate an unacceptable reliability (CV > 10% or ICC < 0.70).

**Fig 2:**
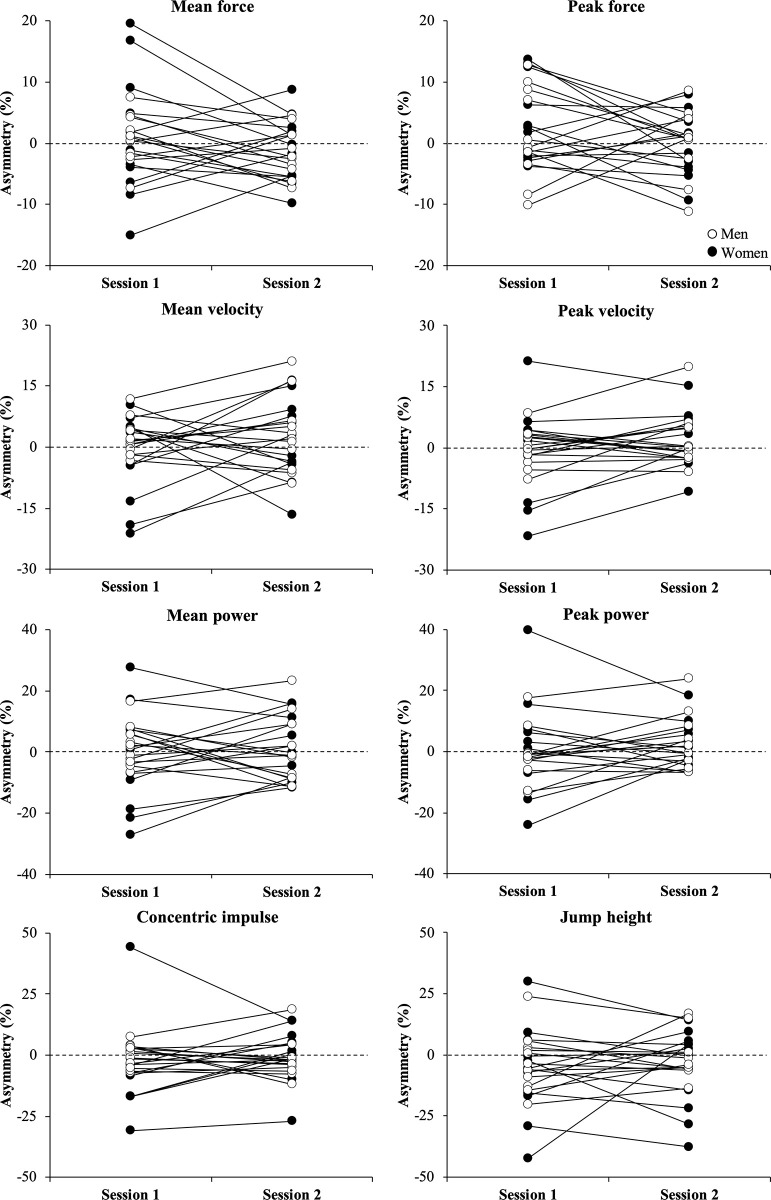
Individual comparisons between testing sessions for the inter-limb asymmetry scores obtained for men (white circles) and women (black circles) during the unilateral countermovement jump exercise.

**Fig 3:**
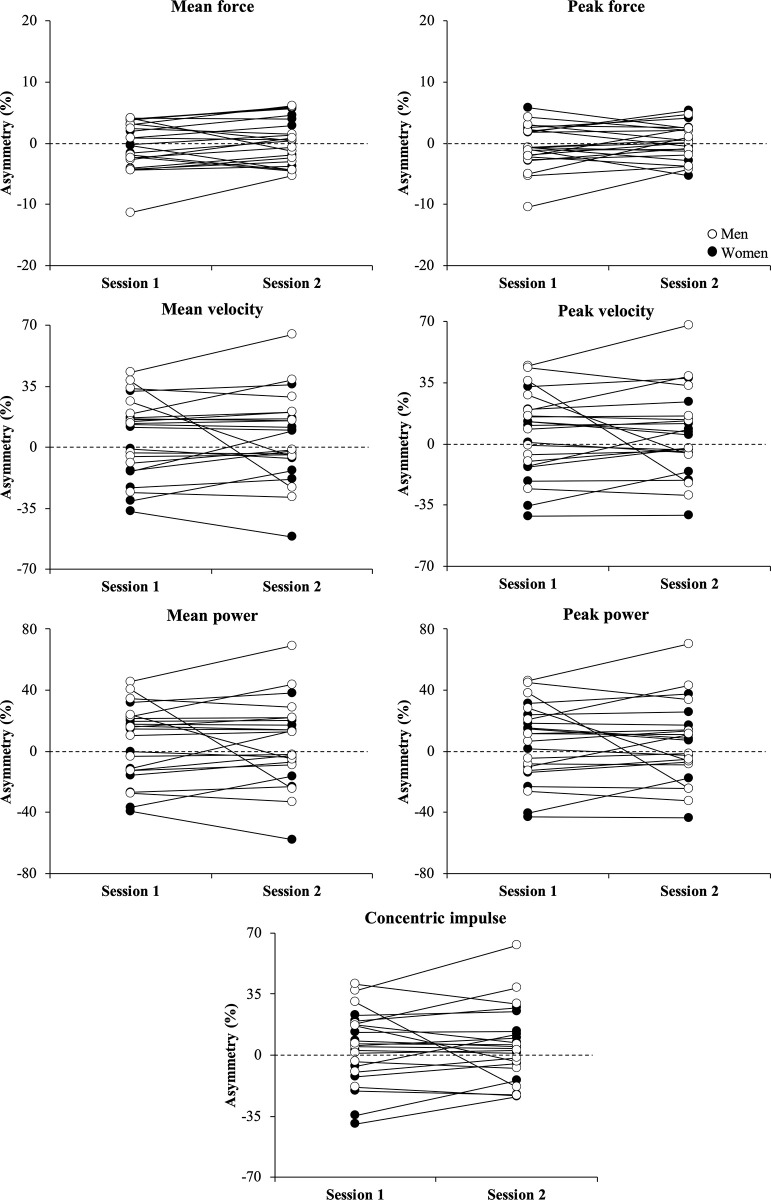
Individual comparisons between both testing sessions for the inter-limb asymmetry scores obtained for men (white circles) and women (black circles) during the bilateral countermovement jump exercise.

Regarding the inter-limb asymmetry variables, no significant differences were observed between sessions (*P* > 0.05) with the magnitude of the differences being either *trivial* (ES ≤ 0.19; 10 out of 15 comparisons) or *small* (0.22 ≤ ES ≤ 0.40; 5 out of 15 comparisons). None of the asymmetry variables met the criterion for acceptable reliability (ICC range = 0.15–0.64) during the unilateral CMJ, while all asymmetry variables reached an acceptable reliability (ICC range = 0.74–0.77) during the bilateral CMJ, with the exception of peak force (ICC = 0.63). Finally, levels of agreement for the direction of inter-limb asymmetries between sessions were from *poor* to *slight* (Kappa range = -0.10 to 0.15) during the unilateral CMJ and *substantial* (Kappa range = 0.65 to 0.74; except for peak force [0.49]) during the bilateral CMJ. Individual comparisons between testing sessions for the inter-limb asymmetry scores are presented in Figs 2 and 3.

## Discussion

This study was designed to compare the between-session reliability of single-leg performance and inter-limb asymmetry variables between unilateral and bilateral CMJ variants. The main findings revealed that i) all single-leg performance variables presented an acceptable reliability with the exceptions of jump height for the unilateral CMJs and mean power, peak velocity, peak power, and concentric impulse for the left leg during the bilateral CMJ, ii) the unilateral CMJ resulted in higher reliability in 2 out of 7 variables for the right leg (mean and peak velocity) and all variables for the left leg (mean and force values of force, velocity, power, and concentric impulse), while the bilateral CMJ was more reliable in 2 out of 7 variables for the right leg (mean force, peak force), iii) the asymmetry variables always showed an unacceptable reliability and *poor*/*slight* levels of agreement during the unilateral CMJ, while an acceptable reliability and *substantial* levels of agreement (except for peak force) were obtained for the bilateral CMJ. These results suggest that single-leg performance can be obtained with higher reliability during the unilateral CMJ, while the bilateral CMJ provides more consistent measures of inter-limb asymmetry.

Single-leg performance evaluation has become increasingly popular in sport and clinical settings in order to provide insight into athletic performance, injury prevention, and rehabilitation [4,5,35,39]. In line with previous studies [5,12–18], our findings revealed that the different single-leg performance variables measured during unilateral and bilateral CMJs present a high between-session reliability, with the exceptions of jump height for the unilateral CMJs and mean power, peak velocity, peak power, and concentric impulse for the left leg during the bilateral CMJ. More importantly, our hypothesis was confirmed since the unilateral CMJ provided a higher reliability than the bilateral CMJ for more variables. These results may be attributed to the variability expected during the weighing phase as a consequence of any postural adjustment prior to jumping [3]. Consequently, the dependent variables of forward dynamics procedures (e.g., velocity and power values) are affected to a greater extent by an inaccurate determination of the body weight during the bilateral CMJ [20]. By contrast, other variables that do not depend on the calculation of body weight, such as mean and peak force, were obtained with higher reliability during the bilateral CMJ [40,41]. These findings are partially in line with Bishop et al. [19] who found a higher reliability (CV_ratio_ = 1.29–1.63) for the bilateral CMJ compared to the unilateral CMJ in single-leg strength performance (mean force and concentric impulse). This result could be caused by the reduction in the base of support during the unilateral CMJ. From a practical perspective, these results generally support the unilateral CMJ as a more cost-effective, reliable and ecologically valid test to measure single-leg performance. The bilateral CMJ can be a more time-effective alternative to evaluate the asymmetries of force within the same repetition, while the interpretation of the rest of the single-leg performance variables should be taken with caution. In addition, since during the bilateral CMJ both legs are in contact with the ground, the bilateral CMJ may offer a better understanding of compensatory strategies between limbs [9].

Despite the growing interest in exploring inter-limb asymmetry within the scientific community [10,21,33], the evidence for the reliability of the asymmetry measurements is still scarce. Previous studies have showed that the inter-limb asymmetry calculated for peak force, jump height, and concentric impulse were highly reliable between sessions (ICC ≥ 0.91) during the unilateral and bilateral CMJs [3,4]. The results of the present study are partially in line with those findings since the relative reliability of the different asymmetry variables was acceptable for the bilateral CMJ (except for peak force). However, the reliability was unacceptable for the unilateral CMJ. These results are probably due to the variable nature in the direction of the asymmetry not only for the leg dominance between metrics and tasks [34], but also for the same test between sessions [8,13]. Specifically, in accordance with the findings of Bishop et al. [8], the direction of asymmetry for jump height performance determined in both CMJ tests varied considerably between sessions. By contrast, Bishop et al. [13] also reported in another study *substantial* levels of agreement for the peak force and jump height asymmetries of the same CMJ exercise. Therefore, the present study confirms the importance of attending not only to the inter-limb asymmetry magnitude, but also its direction as it has been detected as one of the key factors when monitoring inter-limb asymmetries [21]. Although future studies are needed to provide insight into the underlying mechanisms responsible for this varying nature in the direction of asymmetry, the bilateral CMJ seems to be a more consistent test in term of limb dominance to measure inter-limb differences.

Although the current findings provide relevant information about the between-session reliability of CMJ-based tasks to measure single-leg performance and inter-limb asymmetries, this study is not free from limitations. First, although the study sample was accustomed to performing the unilateral and bilateral CMJ, the unfamiliar nature of eliminating the arm swing may have altered the jumping strategy or performance and, ultimately, this would reduce the ecological validity of CMJ testing. However, it has been shown that the reliability of bilateral CMJ performance is even somewhat lower with the use of arm swing in collegiate basketball players [5]. Second, since the inter-limb asymmetries appear to be task-dependent [10], the current findings cannot be generalized to other jumping-based tasks. Future studies should compare the between-session reliability of the performance and asymmetry variables between unilateral and bilateral jumping-tasks performed in other planes of motion (e.g., broad jump).

In conclusion, the between-session reliability for the force platform-based assessment of CMJ performance and asymmetry appears to be task-dependent. On the one hand, the unilateral CMJ is not only a more cost-effective test to measure single-leg performance (only one force platform is needed), but also generally more reliable than the bilateral CMJ. In addition, although the task specificity may ultimately dictate which jump test is chosen, the unilateral CMJ could be a more ecologically valid because most of the sporting actions are performed unilaterally. Instead, the bilateral CMJ is a more time-effective alternative to determine the contribution in force of each limb within the same repetition, but the reliability of the remaining variables is affected to a greater extent by the variability in the weighing phase. On the other hand, the magnitude and direction of asymmetry data was only consistent between-sessions for the bilateral CMJ variant. However, given the variable nature of inter-limb asymmetry, some caution should be taken when interpreting such data.

## Supporting information

S1 Data (XLSX)

## Abbreviations

CMJ: countermovement jump
CV: coefficient of variation
ES: effect size
ICC: intraclass correlation coefficient
SD: standard deviation
